# The Effect of Liraglutide on Epicardial Adipose Tissue in Type 2 Diabetes

**DOI:** 10.1155/2021/5578216

**Published:** 2021-11-16

**Authors:** Na Zhao, Xiaoying Wang, Yongbo Wang, Junjie Yao, Chunhong Shi, Jianling Du, Ran Bai

**Affiliations:** Department of Endocrinology and Metabolism, The First Affiliated Hospital of Dalian Medical University, Dalian, Liaoning Province, China

## Abstract

**Objective:**

To study the effect of liraglutide on the thickness of epicardial adipose tissue (EAT) in type 2 diabetes mellitus (T2DM) patients with abdominal obesity.

**Methods:**

Abdominal obesity T2DM patients with poor glycemic control were collected and treated with liraglutide. The changes of blood glucose, blood lipid, waist circumference, body mass index (BMI), and EAT thickness were compared after 3 months of treatment with liraglutide. Cardiac magnetic resonance imaging (MRI) was used to measure EAT thickness.

**Results:**

After 3 months of treatment with liraglutide, glycosylated hemoglobin (HbA_1c_) decreased from 9.81 ± 1.46% to 6.94 ± 1.29% (95%CI = 2.14–3.59, *p* < 0.001). The weight decreased from 91.67 ± 16.29 kg to 87.29 ± 16.43 kg (95%CI = 2.97–5.79, *p* < 0.001). Waist circumference before treatment was 103.69 ± 9.14 cm, and after treatment was 96.42 ± 8.42 cm (95%CI = 5.04–9.50, *p* < 0.001). Total cholesterol (TC), triglyceride (TG), and low-density lipoprotein cholesterol (LDL-C) were significantly lower than those before treatment. TC decreased from 5.34 ± 1.05 mmol/L to 4.86 ± 0.97 mmol/L (95%CI = 0.15–0.82, *p* < 0.001). TG was 1.89 (1.48-3.17) and then to 1.92 ± 0.69 (*p* = 0.03). LDL-C decreased from 3.39 ± 0.84 mmol/L to 3.01 ± 0.74 mmol/L (95%CI = 0.17–0.59, *p* = 0.001). HDL-C increased by 1.7% after treatment, with no significant difference (*p* = 0.062). More importantly, the thickness of EAT decreased from 5.0 (5.0-7.0) mm to 3.95 ± 1.43 mm (*p* < 0.001) after liraglutide administered for 3 months.

**Conclusion:**

Liraglutide significantly reduces EAT thickness in T2DM with abdominal obesity, which provides theoretical support for the cardiovascular benefits of liraglutide.

## 1. Introduction

Type 2 diabetes mellitus (T2DM) is a metabolic syndrome with disorders of glucose, protein, and fat caused by deficiency of insulin secretion and/or insulin function. It is often accompanied by obesity. Obesity, especially visceral fat accumulation, can lead to islet dysfunction, insulin resistance (IR), prediabetes, T2DM, and inflammatory reaction and increase the incidence of coronary heart disease, hypertension, and other events [1, 2]. Quantification and reduction of visceral fat represent effective methods to identify high-risk individuals and reduce their cardio-metabolic risk of T2DM patients [3]. Epicardial adipose tissue (EAT), as visceral fat, has attracted much attention due to its close relationship with myocardium and coronary artery [4]. The increase of EAT may represent a chronic inflammatory injury and is related to coronary artery disease [5], and T2DM patients had significantly thicker EAT [6]. Liraglutide is a glucagon-like peptide-1 (GLP-1) analog which can activate GLP-1 receptor (GLP-1R). In T2DM patients, studies suggested that GLP-1 receptor agonist (GLP-1 RA) could redistribute adipose tissue deposits and reduce visceral fat [7, 8]. Additionally, liraglutide can reduce the risk of cardiovascular events. Our present study was to explore whether, in addition to its effects on blood glucose and blood lipids, liraglutide reduces cardiovascular events by acting on EAT in T2DM with abdominal obesity.

## 2. Design and Methods

### 2.1. Participant Identification

T2DM patients with abdominal obesity who had poor blood glucose control in Endocrinology Department of Jinpu District, the First Affiliated Hospital of Dalian Medical University from November 2016 to February 2018 were consecutive collected. Participants' inclusion criteria were as follows: (1) aged between 18 and 70 years, (2) body mass index (BMI) > 25 kg/m^2^ and waist circumference ≥ 90 cm in males and ≥85 cm in females, (3) glycosylated hemoglobin (HbA_1c_) was between 7 and 11%, and (4) the basic drugs were metformin and/or *α*-glucosidase inhibitors and/or insulin at the time of enrollment and met the liraglutide indications. Exclusion criteria are as follows: (1) inflammatory bowel disease and diabetic gastroparesis; (2) pregnant or breastfeeding women; (3) moderate to severe liver dysfunction (Child's-Pugh score); (4) acute or chronic renal insufficiency, glomerular filtration rate (GFR) < 60 ml/(min × 1.73 m^2^); (5) history of thyroid tumor or pancreatitis; (6) diabetes with acute complications or with tumor; (7) electronic implants such as cardiac pacemaker, insulin pump, and magnetic metal; and (8) uncontrollable involuntary movement, claustrophobia patients, and so on who are not suitable for MRI examination. With the approval of the medical ethics committee, all participants were informed of the possible side effects of liraglutide and provided informed consent.

### 2.2. Drugs and Dosage

Liraglutide (Victoza, manufactured by Novo Nordisk, Denmark) was injected subcutaneously once a day for 3 months, starting from 0.6 mg/day and increasing to 1.2 mg/day in 3-5 days if there was no adverse. The curative effect of liraglutide was evaluated by monitoring fasting plasma glucose (FPG) and 2-hour blood glucose after three meals every day and before bedtime and early morning if necessary. For patients with poor hypoglycemic effect, the dose was further increased to 1.8 mg/day.

### 2.3. Biochemical Blood Tests

The patients kept a light diet for three days and fasting water for 12 hours before the blood tests. Blood samples were drawn in the next morning. FPG, HbA_1c_, total cholesterol (TC), triglyceride (TG), low-density lipoprotein cholesterol (LDL-C), and high-density lipoprotein cholesterol (HDL-C) were tested ahead of and after 3 months liraglutide being administered by automatic biochemical analyzer (Hitachi 7600, Japan). The 2-hour postprandial blood glucose (2hPBG) was measured by Accu-Chek advantage electronic sensing portable blood glucose meter (Roche, Germany).

### 2.4. EAT Thickness Measurement

EAT thickness was measured before and after 3-month liraglutide treatment by 1.5T cardiac MRI (platform HDxt; General Electric Medical Systems, Waukesha, WI, USA). Scanning parameters were TR 3.6 ms, TE1.6 ms, turning angle 50°, bandwidth 125 kHz, FOV 350 mm∗350 mm, matrix 192 × 224, layer thickness 10 mm, and layer spacing 0. EAT thickness was measured at the right ventricle late diastolic phase with four-chamber echocardiography by two well-experienced radiologists. All cardiac MRI scans were performed by the same radiographer.

### 2.5. Statistical Analysis

The SPSS 19.0 software was used for statistical analysis. Normal distributed data were presented as the mean ± standard deviation (*M* ± SD). Abnormally distributed data were presented as the median (interquartile range). Paired *t*-test and Wilcoxon test were used to compare the differences of data before and after liraglutide treatment. It is considered statistically significant when the *p* value was less than 0.05.

## 3. Results

### 3.1. Subject Enrollment

The basic demographic characteristics of 21 out of 27 subjects who completed the trial are shown in Table 1. After the injection of liraglutide, the patients had varying but tolerable degrees of nausea and loss of appetite. For most of the patients, the above symptoms gradually disappeared without drug intervention after 1-3 days. No severe hypoglycemia was observed during the whole trial, while among the 27 subjects, 5 were withdrawn from the group because of intolerable gastrointestinal reaction, and 1 was withdrawn because of skin allergy at the injection site. The average dose of liraglutide in 3 months was 1.2 mg/day.

### 3.2. FPG, 2hPBG, and HbA_1c_ Decreased

**Figure 1:**
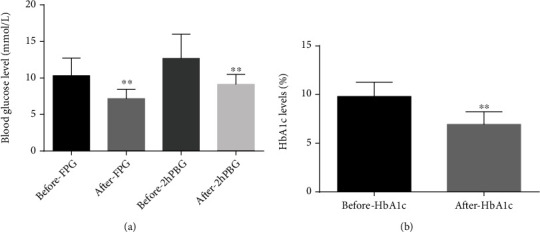
After liraglutide was added, FPG, 2hPBG, and HbA_1c_ decreased significantly. (a, b) FPG and HbA_1c_ were tested by automatic biochemical analyzer, and 2hPBG was measured by Accu-Chek advantage electronic sensing portable blood glucose meter (^∗∗^*p* < 0.01).

After the patients received 3 months of liraglutide, compared with that before liraglutide, FPG decreased from 10.31 ± 2.41 mmol/L to 7.17 ± 1.28 mmol/L (95%CI = 2.07–4.20, *p* < 0.001), and 2hPBG decreased from 12.68 ± 3.33 mmol/L to 9.12 ± 1.37 mmol/L (95%CI = 2.05–5.07, *p* < 0.001), shown in Figure 1(a). What is more, HbA_1c_ decreased from 9.81 ± 1.46% to 6.94 ± 1.29% (95%CI = 2.14–3.59, *p* < 0.001), shown in Figure 1(b). FPG, 2hPBG, and HbA_1c_ were dropped by 30.5%, 28.1%, and 29.3% on average.

### 3.3. Weight, Waist Circumference, and BMI Decreased

**Figure 2:**
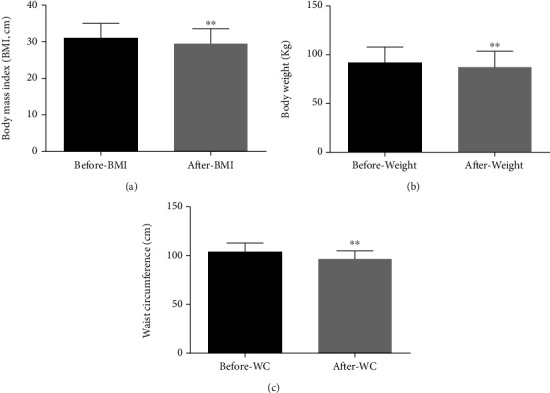
After liraglutide treatment, the weight, waist circumference (WC), and BMI decreased (^∗∗^*p* < 0.01).

The patients' average body weight was 91.67 ± 16.29 kg before liraglutide treatment and 87.29 ± 16.43 kg after treatment, dropped by 4.38 kg on average (95%CI = 2.97–5.79, *p* < 0.001). BMI decreased on average by 1.49 kg/m^2^ from 30.97 ± 4.04 kg/m^2^, and after treatment was 29.48 ± 4.08 kg/m^2^ (95%CI = 1.02–1.98, *p* < 0.001). Waist circumference decreased by 7.27 cm on average from 103.69 ± 9.14 cm to 96.42 ± 8.42 cm (95%CI = 5.04–9.50, *p* < 0.001). Results are shown in Figure 2.

### 3.4. Effect of Liraglutide on Blood Lipid Level

**Figure 3:**
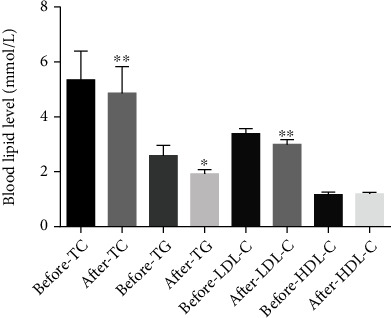
Changes of blood lipids after liraglutide treatment. The levels of total cholesterol (TC), triglyceride (TG), low-density lipoprotein cholesterol (LDL-C), and high-density lipoprotein cholesterol (HDL-C) were measured by automatic biochemical analyzer (^∗^*p* < 0.05 and ^∗∗^*p* < 0.01).

TC decreased from 5.34 ± 1.05 mmol/L to 4.86 ± 0.97 mmol/L (95%CI = 0.15–0.82, *p* < 0.001), TG decreased from 1.89 (1.48-3.17) and then to 1.92 ± 0.69 (*p* = 0.03), and LDL-C decreased from 3.39 ± 0.84 mmol/L to 3.01 ± 0.74 mmol/L (95%CI = 0.17–0.59, *p* = 0.001) (Figure 3). HDL-C increased by 1.7% on average from 0.96 (0.89-1.45) mmol/L to 1.19 ± 0.25 mmol/L, but the difference was not statistically significant (*p* = 0.062).

### 3.5. EAT Thickness Decreased

**Figure 4:**
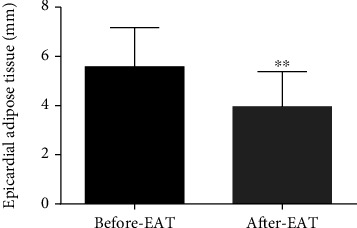
EAT thickness was decreased when liraglutide was introduced. 1.5T cardiac magnetic resonance imaging (MRI) was used to measure the thickness of EAT (^∗∗^*p* < 0.01).

The EAT thickness was 5.0 (5.0-7.0) mm to 3.95 ± 1.43 mm after treatment measured by MRI, with an average decrease of 1.62 mm. The changes suggested that liraglutide can significantly reduce EAT thickness (*p* < 0.001, Figure 4).

## 4. Discussion

Liraglutide, as a GLP-1 analog, has 97% homology structurally with endogenous human GLP-1 [9]. GLP-1 receptor (GLP-1R) mainly exists in fat, liver, gastrointestinal tract, kidney, cardiovascular, and central nervous system [10]. Liraglutide activates GLP-1R to promote insulin secretion of pancreatic *β* cells, reduce body weight, promote vasodilation, and reduce the level of atherosclerotic risk factors in endothelial cells [11–13]. Our results showed that FPG, 2hPBG, and HbA_1c_ of T2DM patients with abdominal obesity were significantly decreased after liraglutide treatment for 3 months, and the body weight, waist circumference, BMI, TG, TC, and LDL-C levels were also significantly decreased. Similar to the results of the present study, liraglutide can effectively reduce HbA_1c_ [14] and body weight [9]. Aoki and his colleagues found that the non-HDL-C and calculated TC were decreased significantly after administrating of liraglutide for 3 months [15]. In addition, in T2DM patients receiving standard care, liraglutide can reduce the risk of cardiovascular events compared with the placebo group [16], but the specific reason is yet not fully clear.

Epicardial adipose tissue (EAT), characterized by brown adipose tissue, is closely connected with the myocardium and coronary artery without fascia barrier. Under physiological conditions, EAT can mitigate the impact, absorb fatty acids, and protect myocardium and coronary arteries from cold injury [17]. It can balance the secretion of proinflammatory and anti-inflammatory factors, regulate the cardiovascular system, and play an important role in obesity-related inflammation and atherosclerosis [18, 19]. Scholars have also confirmed that EAT is positively correlated with obesity, impaired fasting blood glucose regulation, insulin resistance, metabolic syndrome, hypertension, and coronary heart disease through adipocyte hypertrophy and hyperplasia [20, 21]. T2DM patients had significantly thicker EAT associated with cardiac systolic dysfunction [6]. What is more, EAT volume in patients with coronary heart disease with abdominal obesity is significantly higher than that in patients without coronary heart disease [22]. Therefore, for T2DM patients with abdominal obesity, we should not only effectively control blood glucose levels but also monitor EAT levels.

The monitoring of EAT included abdominal circumference, echocardiography, multislice computed tomography (MCT), and magnetic resonance imaging (MRI). Among them, abdominal circumference is the cheapest and most easily obtained visceral fat marker, but it lacks sensitivity and specificity. Echocardiography is noninvasive, reliable, easy to repeat, and relatively accurate, but it cannot obtain a linear measurement value in a single position. Because of its high accuracy, low variability, and high repeatability, MRI results can be used as the reference standard for quantification of EAT and it is considered as the gold standard for measuring visceral fat [23–25]. There are few studies on the measurement of EAT by cardiac MRI. By using cardiac MRI measurements, we found that after 3 months of liraglutide treatment, the thickness of EAT decreased significantly along with the decrease of waist circumference. Similar to the results of this study, Bouchi et al. found that liraglutide can reduce visceral fat and improve the quality of life of diabetic patients [8]. Chinese scholars, in studies of T2DM patients with obesity on the basis of metformin treatment combined with liraglutide, found that it can improve islet beta cell function and significantly reduce visceral fat levels [26]. In addition, some scholars found that receiving metformin for at least 6 months could alleviate the inflammatory burden of coronary perivascular adipose tissue in prediabetic patients and reduce the level of inflammation in coronary artery fat in patients with prediabetes with acute myocardial infarction (AMI) who underwent acute coronary artery bypass grafting (CABG) [27]. Metformin also influences cardiomyocyte lipid. Scholars found that the lipid accumulation and lipotoxic factors of cardiac myocytes were decreased when receiving metformin in hearts transplanted study [28]. Metformin also was used in the basic medication of the present study. Does metformin synergize or enhance the regulation of liraglutide on EAT in this study? However, Iacobellis and colleagues found that there was no EAT reduction in the metformin monotherapy group in subjects with type 2 diabetes at 3 and 6 months study, whereas EAT decreased significantly when treated with additional liraglutide measured by ultrasound [29]. What is more, EAT decreased significantly by GLP-1RA like semaglutide or dulaglutide in a dose-dependent way, and there was no EAT thickness reduction in the metformin group in a 12-week parallel group study [3], and that Jonker and colleagues also found that metformin treatment did not affect the change of pericardial fat (including epicardial fat and paracardial fat) volume [30]. Nevertheless, Ziyrek et al. found that metformin monotherapy for 3 months could reduce the thickness of EAT by echocardiography [31]. What is more, Bizino et al. found that liraglutide mainly reduced subcutaneous fat but not visceral or epicardial fat compared with control groups at a secondary endpoint study [32]. The results were not consistent with the conclusion of the present study. Due to the differences of sample size, race, measuring instrument, follow-up time, and the basic drugs, the results obtained by scholars are inconsistent. We suspect that both liraglutide and metformin are involved in the regulation of visceral fat. Metformin may act on molecular level through the active inflammatory response gradually in visceral fat, while liraglutide can regulate visceral fat more obviously, which was shown in the decrease of EAT thickness in our study. GLP-1R in EAT is related to fatty acid oxidation and white fat browning [33], and there may be a brown fat whitening transition in adipocyte hypertrophy and hyperplasia in EAT of abdominal obesity T2DM patients. The mechanism of liraglutide in reducing EAT considered to be that it reverses the whitening of brown fat in EAT by stimulating GLP-1R after administration of liraglutide, thus reducing the thickness of EAT and protecting cardiovascular. Diabetes patients showed obvious early and progressive myocardial lipid accumulation. Whether or not liraglutide and metformin have the same alleviating effect on diabetic cardiomyopathy need further investigation.

Scholars also found that liraglutide can alleviate endotoxemia-related microvascular thrombosis through GLP-1 receptor signal-mediated immune regulation without affecting blood glucose or HbA_1c_ level, to prevent systemic inflammation, vascular dysfunction, and end organ injury, which has potential clinical significance for the treatment of sepsis [34]. GLP-1R activation reduces vascular inflammation by selectively acting on endothelial cells rather than myeloid cells to reduce the cardiovascular complications of arterial hypertension [35]. Liraglutide could regulate sepsis-induced endothelial dysfunction by reducing vascular inflammation and oxidative stress [36]. Moreover, in the early stage of thrombosis, a specific antithrombotic therapy may inhibit the specific target pathway of coagulation cascade and change the thrombosis process to improve the clinical outcome of patients [37], while it is still unclear and need to be further studied whether and how liraglutide could be used to improve the therapy.

To sum up, this study explored the decrease of FPG, 2hPBG, HbA_1c_, body weight, waist circumference, BMI, TG, TC, and LDL-C levels in abdominal obesity T2DM patients after treatment with liraglutide. Cardiac MRI was used to measure the thickness of EAT. It was found that the epicardial adipose tissue thickness was significantly reduced after 3 months of liraglutide treatment, in addition to effective hypoglycemic and lipid-lowering effects, providing theoretical support that liraglutide could effectively contribute to the protection of the coronary arteries in T2DM patients with abdominal obesity.

## Figures and Tables

**Table 1:** Demographic characteristics of T2DM patients.

Characteristics	T2DM patients
Number	21
Gender (female/male)	6/15
Age (mean ± SD, years)	42.86 ± 11.15
Height (mean ± SD, cm)	171.62 ± 8.49
Weight (mean ± SD, kg)	91.67 ± 16.30
BMI (mean ± SD, kg/m^2^)	30.97 ± 4.04

## Data Availability

All data included in this study are available upon request by contact with the corresponding author.
